# mHealth communication to strengthen postnatal care in rural areas: a systematic review

**DOI:** 10.1186/s12884-019-2531-0

**Published:** 2019-11-06

**Authors:** Florence Mbuthia, Marianne Reid, Annali Fichardt

**Affiliations:** 1 Dedan Kimathi University of Technology, Kenya, PO Box 657-10100, Nyeri, Kenya; 2 University of the Free State, PO Box 339, Bloemfontein, 9300 Republic of South Africa

**Keywords:** Postnatal care, Mobile health, Communication, Rural, Systematic review

## Abstract

**Background:**

Postnatal care (PNC) in rural areas is characterised by low uptake, with possible effect on maternal and neonatal mortality rates. Mobile health (mHealth) communication has been proposed to promote the uptake of health services; however, there is limited information on how mHealth can strengthen PNC in rural areas. The objective of this review was to gather the best available evidence regarding mHealth communication to strengthen PNC in rural areas.

**Methods:**

Studies published between 1 January 2008 and 31 August 2018 were searched in electronic databases hosted by EBSCO Host. Reference list checking and contact with authors were also done. Critical appraisal of the eligible studies was also done.

**Results:**

The results of 11 articles were synthesised to report the determinants of PNC uptake. Determinants were aligned to the Integrative Model of Behaviural Prediction (IMBP). One-way mobile phone messaging was the most common type of mHealth communication used. mHealth communication influenced mothers’ intentions, skills, and environmental constraints associated with uptake of PNC. Intentions were influenced by attitudes, perceived norms and self-efficacy. Positive attitudes, as well as changed attitudes toward PNC practices were observed. Perceived norms that were enhanced were delivery at a health facility with immediate PNC, seeking of reinforcement and professional health support of newborn care practices, and male partner support. Improved self-efficacy was demonstrated by mothers who attended scheduled appointments and they were confident with regard to newborn care practices. Skills for PNC that were improved included cord care, thermal care, appropriate breastfeeding and problem-solving. The environmental constraints faced and which were addressed in the studies included inaccessibility, unavailability and unaffordability of PNC services in rural areas.

**Conclusions:**

Results from the literature included in this study show that one-way mobile phone messaging is the common type of mHealth communication used to strengthen PNC in rural areas. mHealth communication can influence intentions, skills and environmental constraints as determinants of PNC uptake. mHealth communication is recommended to strengthen PNC in rural areas. To widen the evidence, more studies in the field of mHealth communication that report a variety of both maternal and neonatal outcomes are needed.

## Background

Postnatal care (PNC) is the care provided to the mother and the neonate from birth up to 6 weeks [1]. This study focused on the PNC care provided by health professionals. PNC improves the chances of survival of both the mother and the neonate, by helping prevent and treat complications arising from childbirth, and by providing the mother with valuable information on how to care for herself and her neonate [2]. PNC care is recommended to improve maternal and neonatal health, which has received relatively little attention in many low- and middle-income countries (LMICs) [3].

Globally, the uptake of PNC shows a substantial gap between LMICs and high-income countries, with high-income countries reaching 90% of mothers and neonates, compared to only 30% being reached in LMICs [4]. PNC that is provided in LMICs tends to be lower and weaker in rural areas than in urban areas; this phenomenon creates regional inequalities, and holds disadvantages for the population living in such areas [5]. Rural areas refers to areas of open country and small settlements [6]. Rural areas are characterised by poor access and uptake of health services, in addition to other issues, such as low literacy levels, large geographical areas, social marginalisation, unskilled human resources and inadequate financial resources among the population [7, 8]; all these factors can influence uptake of PNC services. For the purposes of this study, all areas outside an urban area were considered to be rural. Among the LMICs that report suboptimal uptake of PNC in rural areas are Kenya and Nigeria. These two countries have substantial urban-rural disparities: the uptake in rural Kenya is 49%, compared with 72% in urban areas [9], while Nigeria reports a PNC uptake of 27% in rural areas, compared to 73% in urban areas. Tanzania also experiences this disparity, with less than 25% of the population in rural areas utilising PNC [10]. To address this inequality, and to strengthen PNC in rural areas, practical solutions have to be devised.

Technological approaches to solving this problem, such as mobile Health (mHealth), have the the potential to reduce inequalities in PNC uptake by improving communication between healthcare providers and clients [11]. Studies generally propose mHealth communication interventions as effective solutions for problems relating to maternal and neonatal health [12, 13]. These interventions are recommended to enhance preventive maternal and healthcare services by improving client education and behaviour change communication, tracking of registries/vital events, data collection and reporting, provider-to-provider communication, and electronic health records [14, 15]. All these aspects are essential for strengthening uptake of PNC, particularly in rural areas where accessibility of maternal and neonatal health often has numerous obstacles, such as social, geographical and economic barriers, shortages of skilled health staff, lack of information on sexual and reproductive health, the cost of medical treatment, sociocultural factors and negative perceptions of quality of care [16, 17].

The increasing use of mobile phones by diverse populations holds promise that implementing mHealth interventions could expand healthcare coverage and strengthen the uptake of health services, such as PNC [18]. The availability and, thus, utilisation of mobile phones is growing rapidly, almost the entire world population, or 96%, is now living within reach of a mobile cellular network, with developing countries registering much faster growth in mobile broadband subscriptions than developed countries [19]. Though rural areas are said to be limited regarding mobile penetration compared to urban areas [20], the increasing trend in penetration has great potential for the application of mHealth interventions in rural areas.

Though these interventions have been proposed to address a variety of health challenges, there is limited literature and evidence to demonstrate how mHealth interventions can be used to strengthen PNC in rural areas. The World Health Organization has identified knowledge gaps in the area of mHealth, and recommends more research to be undertaken to evaluate the role of mHealth interventions in the delivery of PNC, and to determine how the interventions could contribute to improving maternal and perinatal health [1]. The objective of this study was to perform a systematic review of the best available evidence about mHealth communication, in order to strengthen PNC in rural areas. The Integrative Model of Behavioral Prediction (IMBP) [21] was used in this review to highlight various determinants that may strengthen PNC uptake.

### Theoretical framework

**Fig. 1:**
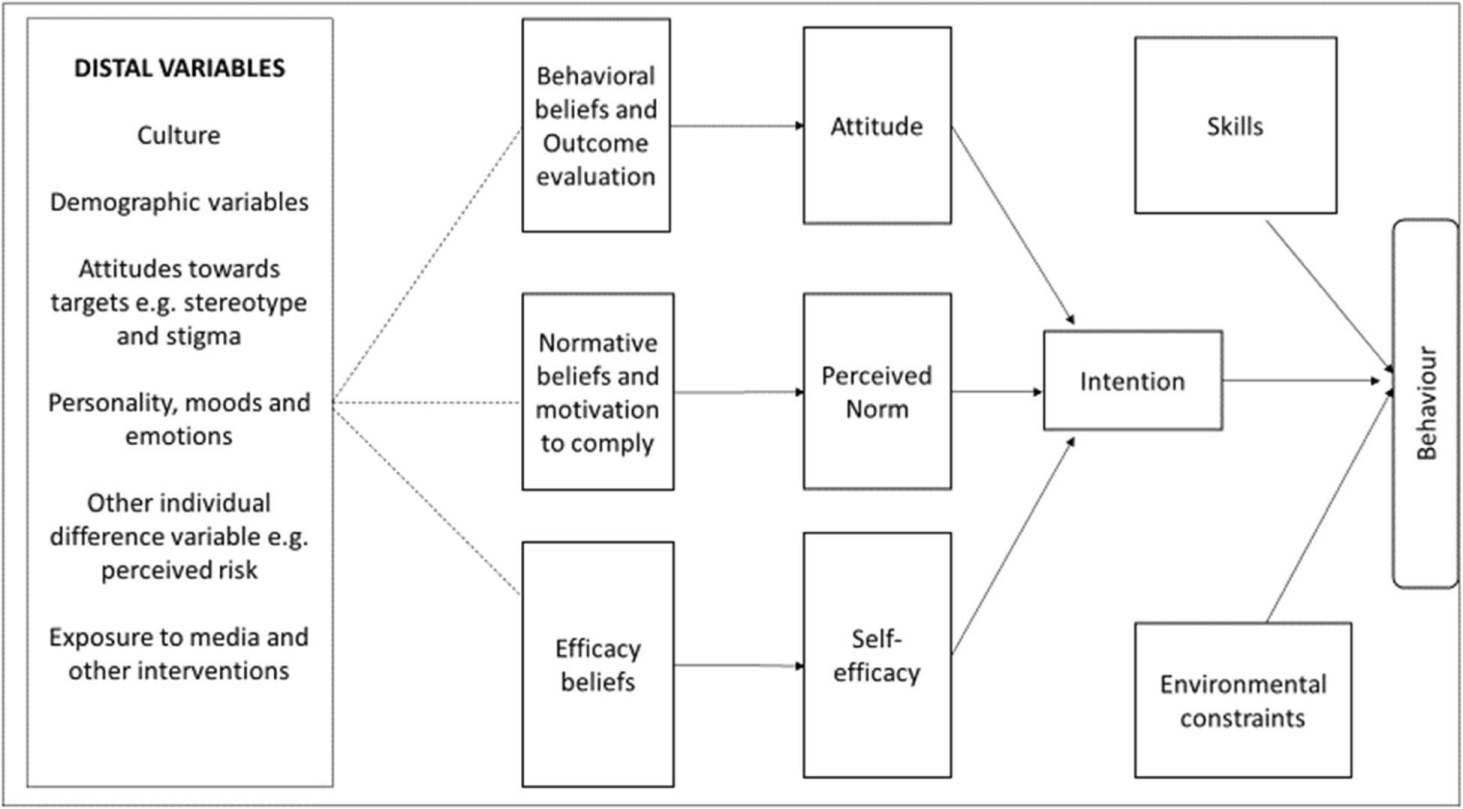
An integrative model of behaviour predictio n[21]. Dashed arrows indicate that the background variables do not always, and in the same manner, shape beliefs

The IMBP (Fig. 1) guided this review by identifying the determinants of behaviour change that might affect PNC uptake. The IMBP accounts for uptake of any behaviour in any given population [22]. It is a powerful model for predicting behaviour across a variety of fields, and has been used to boost or reduce certain behaviours with public health implications [23–26]. According to the IMBP, only a small number of determinants need to be considered to predict, change, or reinforce a particular behaviour in any given population [22]. The model proposes that any given behaviour is most likely to occur if a person has a strong intention to perform the behaviour, if a person has the necessary skills and abilities required to perform the behaviour, and if no environmental constraints prevent behavioural performance [21]. Intentions are influenced by the attitude toward performing the behaviour, perceived norms concerning performing the behaviour, and the person’s self-efficacy with respect to performing the behaviour [21, 22]. Attitudes, perceived norms, and self-efficacy are global perceptions that represent a variety of specific beliefs about the particular behaviour [22]. They are all functions of specific beliefs that influence intentions in the model. In this review, the uptake of PNC was determined by highlighting the various outcomes aligned with the primary determinants of the behaviour in the model, i.e., intention, skills, and environmental constraints.

## Methods

Preferred Reporting Items for Systematic Review and Meta-Analysis (PRISMA) guidelines were followed in this systematic review [27]. The PICO (population, intervention, comparison, outcome) format [28] was used to develop a research question that guided the search strategies and review, namely, how can mHealth strengthen PNC in a rural area? The protocol was registered in the International Prospective Register for Systematic Reviews (PROSPERO) CRD42018102299 [29].

### Search strategies

After a rapid appraisal, search words were generated from three major PICO elements (population, intervention, outcome) and a group of synonyms and related concepts was created and combined using OR and AND operators, to ensure comprehensive search (Table 1). The following databases were used on the EBSCOHost interface: Academic Search Complete, Africa-Wide Information (containing data from the National Research Foundation database of current and completed research projects), CAB Abstracts, CINAHL with Full Text, Communication & Mass Media Complete, ERIC, Health Source: Consumer Edition, Health Source: Nursing/Academic Edition, MasterFILE Premier, MEDLINE with Full Text (containing the same data as PubMed), PsycINFO, SocINDEX with Full Text and SPORTDiscus with Full Text. In addition, checking the reference lists of the studies that were included added three further studies. The authors were contacted in the case of two studies, to confirm the settings of the studies. Before the final analysis, the search was rerun, but no other studies were retrieved for inclusion.

**Table 1:** Search words aligned to PICO

Variable	Search Words
Population: Postnatal mothers or involved community members (any person that could support the mother/baby to ensure better PNC outcome, e.g., father; grandmother; village elder; community health worker)	(family* or families* or mother* or father* or parent* or (communit* n3 (carer* or caregiver* or care-giver* or “care giver*” or “lay worker*” or “health worker*” or volunteer*) (child* or pediatr* or paediatr* or postnatal* or post-natal* or neonat* or newborn* or “after birth*” or “MATERNAL health service*”)
Intervention: Mhealth intervention	AND (mHealth or “mobile health” or sms or “mobile phone*” or “mobile telephone*” or cellphone* or “cell phone*” or “text* messag*” or “short message service*“or mHealth or m-health or “mobile health” or ehealth or e-health or sms* or “instant messag*” or “mobile phone*” or “mobile telephone*” or cellphone* or “cellular phone*” or “cellular telephone*” or smartphone* or “smart phone*” or “mobile device*” or “electronic device*” or “portable device*” or “phone intervention*” or “telephon* intervention*“or online or app or apps or “WIRELESS communication systems in medical care” or “ONLINE information services”)
Outcome: beliefs, attitude; norms; self-efficiency; skills; intention; environmental constraints	(belief* or conviction* or faith or trust* or norm or norms or custom* or attitude* or outlook* or approach* or Self-efficac* or ability* or Skill or skills or expertis* or ability* or talent* or proficien* or knowhow or capability* or knack or competent* or Intent* or determination* or planning or resolve or decide* or decision* or choose or select* or choice*)

### Study selection

**Fig. 2:**
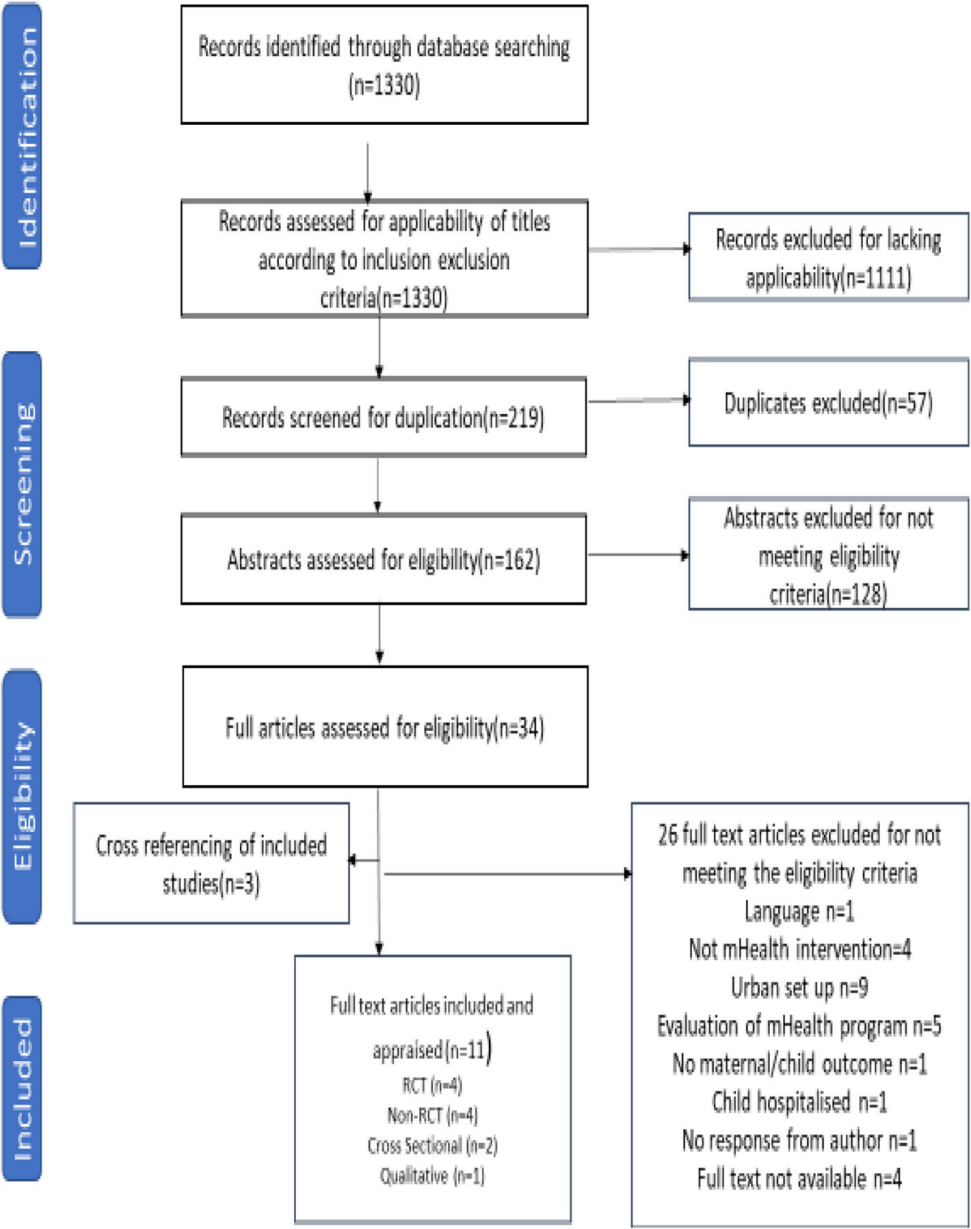
PRISMA flow diagram for database search of studies. The diagram show the studies included and reasons for excluding some of the studies

The inclusion criteria that were applied included that the literature had to be written in English, or that studies written in other languages had to have abstracts written in English, mHealth communication had to be between healthcare personnel and mothers, or involved community members, mHealth communication was aimed at improving health outcomes of either a mother or child, mHealth communication was with mothers or community members from semi-urban and rural areas, and that literature had to have been published between 1 January 2008 and 31 August 2018. Exclusion criteria were that mHealth communication focused on peer support amongst mothers, mHealth communication was with mothers or community members whilst mothers were still hospitalised, literature involved evaluation of mHealth programmes and health systems, and literature represented research briefs, research news, commentaries, editorials or proposals. Three reviewers (FM, MR and AF) independently screened titles, abstracts, and full-text articles to decide whether an article was relevant. Any discrepancies among reviewers were resolved by obtaining consensus. Only studies published in peer-reviewed journals were included. The results of study screening and selection are illustrated in Fig. 2.

### Data extraction

A standardised, pre-piloted form was used to extract data from the studies included, to assess study quality and to synthesise evidence. Extracted information included bibliographical details, methodology, outcomes and intervention, critical appraisal rating and study findings (see Additional file 1.).

### Quality assessment of studies included

Three reviewers conducted the critical appraisal of the study independently, to ensure the validity and to increase the reliability of the process. A critical appraisal checklist that was suitable for each research design of studies that were appraised was selected. For randomised, controlled trials and qualitative and cross-sectional studies, checklists developed by the Critical Appraisal Skills Programme (CASP) [30] were used to assess the quality of each study. The quality of non-randomised studies was examined using the checklist for quasi-experimental studies (non-randomised experimental studies) of the Joanna Briggs Institute [31]. Although the scores differed according to the research design used, all these checklist tools focused on the following aspects: appropriateness of methods used, clarity of focus, the recruitment process, the accuracy of measures used, data collection, presentation and analysis, clarity in the statement of the findings, and appropriateness of context. Higher scores indicated higher quality. A data extract sheet provides the quality assessment of all studies included. Grades were assigned to show the level of the evidence according to guidelines of the American Dietetic Association [28] (Additional file 1).

## Results

A narrative synthesis of the results of the studies, structured around IMBP, was done to highlight the best evidence of mHealth communication for strengthening PNC in rural areas. Results were gathered from the 11 articles that met the eligibility criteria. The results summarise the type of mHealth communication used and determinants that strengthen PNC, namely, intentions, skills and environmental constraints. Intentions were influenced by attitudes, perceived norms, and a person’s self-efficacy with respect to PNC uptake (Additional file 2).

### Types of mHealth communication used to strengthen PNC in rural areas

Seven types of mHealth communication were used to strengthen PNC in rural areas; these were phone calls, one-way messaging, interactive messaging, audiovisual material and videos, voice messages, and combined messaging and phone calls. Mobile phone calls were used by village health team members to consult healthcare professionals on matters regarding PNC [32, 33]. These calls were also used by healthcare practitioners (HCPs) to remind mothers to attend PNC clinics and to inform them of their laboratory results [7, 13, 34, 35]. One-way mobile phone messaging was used by HCPs to remind mothers of clinic appointments, communicate test results from the clinic and to give them information on PNC [7, 13, 34–39]. Interactive mobile phone messaging between HCPs and mothers was used to discuss breastfeeding practices and remind mothers to attend PNC clinics [40]. Mobile phone audio visual application and educational videos was sent to mothers by HCPs to illustrate various PNC practices [37]. A combination of one-way mobile phone messaging and phone calls was used by HCPs to remind mothers about their clinic appointment dates [13, 35]. Voice messages were delivered to mothers by HCPs to inform them of neonatal care [7]. Among all these types of mHealth communication, one-way mobile phone messaging was commonly used to influence the three primary determinants of PNC uptake, i.e., intention, skills and environmental constraints.

### Intentions

Intentions, as described in the IMBP, are influenced by attitudes, perceived norms and self-efficacy, all of which are functions of specific beliefs described in the model. Though not all articles addressed the three determinants of intention, the themes emerging from the results that were found to change or maintain PNC outcomes targeting attitude, perceived norms, and self-efficacy, were as follows.

#### Attitudes

Although not all the articles addressed attitude change, some studies indicated that positive attitudes were observed, while other studies specified changes in attitudes of mothers when mHealth communication was used. In Kenya, some mothers changed attitudes towards attending postnatal clinics for HIV testing of their infants and, as a result, their infants were tested [40]. In rural areas of Guatemala, some mothers changed their attitudes to exclusive breastfeeding after health professionals wrote text messages to them on newborn nutrition and, consequently, they started breastfeeding exclusively [38]. In rural areas of Ethiopia, there were postive attitudes observed about delivering in the hospital and seeking PNC services in health centres. In rural areas of Bangladesh, some mothers changed their beliefs about PNC and consequently attended PNC clinics to have their children vaccinated in a timely manner [39], they changed attitudes to recommended PNC practices, such as delaying the first bath and breastfeeding babies immediately after birth [7]. In rural areas of Uganda, providing additional information through mHealth communication lead to attitudinal change on newborn care practices among some mothers, i.e., breastfeeding immediately after birth, cord care and thermal care by mothers [32].

#### Perceived norms

The results report enhanced perceived norms about the uptake of PNC services when mHealth interventions were used. For example, in rural areas of Uganda, Ethiopia and India, HCPs used mHealth communication to influence mothers to deliver in hospitals and seek care for newborns’ illnesses. Previously, the norm in these countries had been to deliver at home, and mothers delayed seeking care for newborns’ illnesses [33, 36, 37]. In Ethiopia, mHealth interventions influenced mothers to deliver in the presence of trained health extension workers and to use PNC immediately; this represented an improvement in the use of PNC compared to their intial preference for delivering at home and not using PNC soon after delivery [36]. In rural areas of Uganda, use of mHealth communication with health professionals during home visits elicited male partner participation in PNC issues; these men were not previously involved, and their participation enhanced uptake of PNC [32].

#### Self-efficacy

Although not all articles addressed self-efficacy, the results show improved self-efficacy with the use of mHealth communication among some mothers. Postnatal women in particular, demonstrated a capacity to adhere to recommended PNC practices. In Cameroon, some mothers gained the confidence to attend scheduled PNC appointments for HIV care [34]. In India, mothers demonstrated the ability to recognise and report complications during the postnatal period [37]. In Guatemala, the use of mHealth communication enhanced mothers’ capacity to breastfeed their newborns exclusively [38]. In Ethiopia, mHealth communication assisted mothers to become receptive to following PNC advice [13]. In Uganda, use of mHealth communication helped mothers to have confidence that, by observing recommended newborn practices, their babies would achieve favourable outcomes, such as proper cord healing [32].

### Skills

Though it was not reported by all studies that were reviewed, several skills for PNC were strengthened. mHealth interventions such as reminders to mothers on skills previously taught were used. In Uganda, mothers gained skills in cord care, thermal care and breastfeeding immediately after birth when health professionals explained the skills through mobile phone consultations [32, 33]. When text messages were sent to mothers in Guatemala, providing information regarding breastfeeding practices, mothers reported that they practiced exclusive breastfeeding successfully [38]. In Bangladesh, when text messages were used to give information on neonatal care, mothers demonstrated skills related to breastfeeding immediately after birth and bathed newborns after 3 days [7].

### Environmental constraints

mHealth communication addressed possible environmental constraints, such as inaccessibility, unavailability and affordability, which act as barriers to the uptake of PNC services in rural areas, although not all the articles explored this aspect. For instance, mHealth communication addressed the inaccessibility of PNC in rural areas of Uganda, and helped mothers to access information in a timely fashion, and prompted maternal and newborn care [32, 33]. In rural areas of Ethiopia, mHealth communication was used to track mothers by notifying them of PNC services; this provided information on the availability of PNC services [13, 36]. In Kenya, mothers reported that receiving mHealth communication facilitated PNC services [40]. In India, text messages to mothers facilitated more significant contacts without requiring mothers to travel to a health facility, and this enhanced the availability of PNC services [37]. With the use of mobile phone messaging to track newborns due for vaccination, there was timely vaccination of children in rural areas of Bangladesh – the messaging facilitated PNC [39]. In Zambia, text messaging was used to encourage mothers to return to the clinic and to give test results, leading to early diagnosis and enrolment for care [35].

## Discussion

Results from the data synthesis give evidence that a variety of mHealth communication can be used to strengthen PNC in rural areas. One-way mobile phone messaging is the most common type of mHealth communication that is used to strengthen PNC uptake in various rural areas. This is a clear indication that mobile messaging may be the communication of choice for most users instead of other means of mHealth communication. This inference is in line with findings of studies done by Feroz et al. and Watterson et al. [14, 41], who found improved uptake of health services when mobile messaging was used, compared to other types of mHealth communication. Mobile messaging has been cited to be cost effective to implement, and is rated highest for achieving favourable outcomes in health programmes [42–44].

To help with interpretation of the results, the IMBP was used to describe determinants of PNC uptake in rural areas. The evidence from the results indicate that the intention to use PNC services was influenced by positive or changed attitudes, enhanced perceived norms, and improved self-efficacy. Mothers acquired various skills that are required during the postnatal period, and which facilitated use of PNC. Also, the application of mHealth communication reduced environmental constraints that usually hinder the uptake of PNC. All these changes lead to uptake of PNC in rural areas.

Attitudes of mothers to PNC uptake changed in some studies, while others reported positive attitudes with the use of mHealth communication; this attitude was towards PNC practices, such as breastfeeding, cord care, thermal care and care-seeking on newborns’ illnesses. A study in Sierra Leone reports that mHealth communication helped mothers to change their attitudes to family planning and, as a result, there was increased uptake of contraceptive services [45]. A study in Malawi reported a change in attitude about immunisation among mothers who received mHealth communication via SMS [43]. Though the two studies in Sierra Leone and Malawi reported on PNC services, these studies were excluded from the systematic review of the current study, since health system programmes were evauated, rather than mHealth interventions. Furthermore, a positive attitude to PNC services has been recognised as a key factor in the uptake of services [46–48].

From the results it is clear that mHealth communication also enhanced perceived norms. Accordingly, mothers utilised immediate PNC, and received support and reinforcement from HCPs and mothers’ male partners. Adopting norms, such as hospital delivery, leads to uptake of PNC services. One study demonstrated that women who deliver in a hospital are more likely to use PNC than those who deliver at home [49]. mHealth communication has been identified as a catalyst for involving male partners and gaining their support for uptake of maternal and neonatal health services [50]. These norms are among those recommended by the World Health Organization for uptake of PNC services [1].

The results highlight improved self-efficacy of mothers, who became confident with PNC services and were able to use them when mHealth communication was used. Mothers expressed confidence about following advice, and reported active coping when caring for their newborns. When HCPs used mHealth communication, clients in Cambodia reported improved self-efficacy in using contraception [51]. Other studies have reported improved self-efficacy, although this self-efficacy was for health workers who used mHealth communication [52–54].

This review shows that exclusive breastfeeding, thermal care, cord care and problem-solving skills were strengthened by mHealth communication. Other studies report similar findings, though in different contexts. For example, exclusive breastfeeding and problem-solving skills were strengthened when mHealth communication was used by HCPs in one of the most urban areas of the United States [55]. There are not many studies that report on skills improvement among clients who use mHealth communication, but Labrique et al. [56] suggest that mHealth communication can reinforce and monitor skills among HCPs. In Uganda, mHealth communication improved the skills of community health workers involved in the programme implementation and who were required to manage newborns’ illnesses [54].

Environmental constraints, which are commonly cited as a hindrance for the uptake of any health services, were also addressed, and evidence suggests that mHealth communication assists to improve accessibility and availability and promote affordability of PNC services in rural areas. These findings are in line with other studies that demonstrate that mHealth communication reduces physical barriers to accessibility [14, 49, 54] and avails health services at reduced costs [57]. Furthermore, uptake of maternal care is hindered mainly by poor geographical access [16].

This systematic review had its strengths and weaknesses. To strengthen the review, the IMBP was used to help synthesise evidence on specific determinants that lead to PNC uptake. The reviewer also used thorough and clear inclusion and exclusion criteria. The study critically appraised papers published in peer-reviewed journals. The inclusion of five randomised controlled trials strengthened the evidence presented herein further. The quality of studies was rigorously determined and most of them scored highly. Although the articles that were included were few in number and heterogeneous, there is evidence that mHealth communication can be used to strengthen PNC in rural areas and should, therefore, be utilised, especially considering the advances of mHealth technology.

Nevertheless, the study had limitations. Only papers written in English were eligible for the review and the studies included had limited PNC outcomes that were related to mothers. The term “postnatal” as recommended by WHO was used, rather than “postpartum”, which possibly explains the the limited outcomes related to mothers. Not all studies reported on every IMBP determinate or only reported positive outcomes. The authors acknowledge that not all rural areas reported on in this study are similar.

## Conclusion

This systematic review concludes that, although a variety of mHealth communication types can be used to strengthen PNC in rural areas, the commonly used type was one-way mobile messaging. mHealth interventions can improve uptake of PNC by influencing intentions and skills and reducing environmental constraints. By changing beliefs related to attitudes, perceived norms and self-efficacy, intentions to use PNC are enhanced. Skills for PNC that can be strengthened by use of mHealth communication are breastfeeding, cord care, thermal care, delayed bathing of babies, safer sleep practices, care-seeking and problem-solving. mHealth communication can reduce environmental constraints, such as inaccessibility, unavailability and unaffordability of PNC services in rural areas, thus, facilitating uptake of these services. Use of mHealth communication is, therefore, recommended to strengthen PNC in rural areas. To strengthen the evidence, there is a need for more studies in the field of mHealth communication that report a variety of both maternal and neonatal outcomes.

## Supplementary information

**Additional file 1.** Data extraction and critical appraisal of studies included. This file provides bibliographic details, and information on the aims, design, setting, sample, inclusion-exclusion criteria, primary and secondary outcomes, data collection, intervention, control group, data analysis, critical appraisal, study findings, limitations and recommendations of each article.

**Additional file 2.** Data synthesis on how mHealth strengthens PNC in a rural areas. This file highlights the types of mHealth communication and the determinants of PNC uptake.

## Data Availability

The datasets used and/or analysed during the current study are available from the corresponding author on reasonable request.

## Abbreviations

CASP: Critical appraisal skill programme
HCP: Health care provider
IMBP: Integrative model of behavior prediction
JBI: Joanna Briggs Institute
LMICs: Low and middle-income countries
mHealth: Mobile health
PICO: Population, intervention, comparison, outcome
PNC: Postnatal care
PRISMA: Preferred reporting items for systematic review and meta-analysis
PROSPERO: Prospective register for systematic reviews
RCT: Randomised controlled trial
WHO: World Health Organization
