# Supplementary material for: mHealth communication to strengthen postnatal care in rural areas: a systematic review
Source: BMC Pregnancy Childbirth. 2019 Nov 6;19:406. doi: 10.1186/s12884-019-2531-0 (PMC6836428; doi:10.1186/s12884-019-2531-0)
Supplement: Supplementary file 1 — Additional file 1. Data extraction and critical appraisal of studies included. This file provides bibliographic details, and information on the aims, design, setting, sample, inclusion-exclusion criteria, primary and secondary outcomes, data collection, intervention, control group, data analysis, critical appraisal, study findings, limitations and recommendations of each article. [file 12884_2019_2531_MOESM1_ESM.docx]

**Additional file 1: Data extraction and critical appraisal of studies included**

| **No.** | **Bibliographic details** | **Methodology** | **Outcome and intervention** | **Critical appraisal/ level of evidence** | **Study findings** |
| --- | --- | --- | --- | --- | --- |
| Randomised controlled trials(n=4) | | | | | |
|  | Ayiasi RM, Kolsteren P, Batwala V, Criel B: **Effect of village health team home visits and mobile phone consultations on maternal and newborn care practices in Masindi and Kiryandongo, Uganda : A community-intervention trial**. *PLoS Med* 2016 **11**(4):e0153051. doi: 10.1371/journal.pone.0153051 | Aim: To measure the effect of home visits combined with mobile phone consultations on maternal and newborn care practices. Design: Randomised controlled trial Setting: 16 health centres in Masindi and Kiryandongo districts, Uganda. Sample: 1 388 participants from 16 clusters Inclusion criteria: First antenatal visit.  Cut-off of 28 weeks of gestation. Exclusion criteria: Women above 28 weeks of gestation. | Primary outcomes: To increase health facility delivery. Secondary outcomes: To improve maternal outcomes related to   - antenatal clinic attendance; and - birth preparedness.   To improve newborn outcomes related to   - cord care; - thermal care; - breastfeeding practices; and - care-seeking for newborn illness.  Data collection: During recruitment, biodata information was recorded.  After the intervention, 10 research assistants and three supervisors collected data using structured questionnaires.  Data was collected on maternal practices:   - Antenatal attendance - Birth preparation - Place of delivery;   And newborn care practices:   - Tying and cutting of the cord; - Wrapping and bathing of the newborn; - Initiation of exclusive breastfeeding; and - Care-seeking in case of newborn falling ill.  Intervention group: Village health teams (VHTs) were given mobile phone handsets capable of making unlimited phone calls for consultation with professional health workers.  The VHTs made two prenatal and one postnatal home visit to households to provide educational messages for maternal and newborn care. Control group: No mobile phones.  No follow-up visits by VHTs.  Routine education in the health centres. Data analysis: Chi-square test statistic to compare individual characteristics.  Multi-variable analysis for covariates that showed significant differences.  Cluster-level analysis to cater for intra-cluster correlation.  Odds ratios using random effects. | Critical appraisal tool used: Critical Appraisal Skills Program (CASP) tool for randomised controlled trial.  Randomisation rigorous.  Participants managed rigorously.  Attrition low.  Data analysed according to groups.  Groups and interventions comparable.  Context appropriate. Score on appraisal: 10/11  Included  Level of evidence: II | Primary outcomes: Health facility delivery 90% in intervention compared to 28% in control arm. This was statistically significant [17.94 (6.26–51.37); p<0.001]. Secondary outcomes: Antenatal visits by women were 85% in the intervention compared to 71% in control – not statistically significant.  Birth preparation was adequate in 51.8% of women in intervention group compared to 20.8% among the control. This was statistically significant [2.58 (1.00–6.65); p=0.05]  Neonatal outcomes showed significant associations with the intervention, except for breastfeeding practices.  Cord care [3.05 (1.81–5.12); p<0.001]  Thermal care [7.58(2.52–22.82); p<0.001]  Breastfeeding practices [71.26 (0.70–2.29); p=0.44]  Timely care-seeking care for newborns’ illnesses [4.93 (1.59–15.31); p=0.006] Limitations: Health worker attrition delayed the recruitment of study participants.  Midwives had to attend workshops frequently, causing further delays in the recruitment process. Recommendations: Further investigations on community problems, such as offering pre-lacteal feeds and early bathing of newborns, to identify appropriate solutions. |
|  | Atnafu A, Otto K, Herbst CH: **The role of mHealth intervention on maternal and child health service delivery: findings from a randomized controlled field trial in rural Ethiopia**. *mHealth* 2017, **3**:39–39. doi: 10.21037/mhealth.2017.08.04 | Aim: To determine whether a locally developed mobile phone SMS-based data exchange application influencing health extension workers can improve maternal health services, contraceptive utilisation rates, and immunisation coverage compared to a traditional approach. Design: Randomised controlled trial Setting: Three districts in rural Ethiopia. Sample: 1 080 mothers/district Inclusion criteria: ***For baseline survey:*** Women 15–49 years. Women with child younger than than 5 years.  ***Post intervention survey:***  Women 15–49 years.  Woman with child younger than one year old. Exclusion criteria: Not stated | Primary outcomes: To find out:   - % change in ANC attendance: timing and number of visits; - % change in clean and safe deliveries; - % change in deliveries attended by skilled provider; - % change in stock-out of contraceptives at the health post level; - % change in immunisation coverage (TT2, Penta1, Penta3, measles); and - % of women visited by a health education worker during pregnancy.  Secondary outcomes: Not stated Data collection: Data was collected on:   - ANC service utilisation; - Delivery services; - Family planning utilisation; - Child immunisation; and - PNC visit by community health worker.   The survey was conducted twice, once at baseline and at the end of the intervention.  Pre-tested, structured questionnaire was used by a team of data collectors that included a district field supervisor, local supervisor and data collectors at each district Intervention group: There were two intervention groups.   - Treatment 1 partial intervention: all HEWs received a mobile phone with a customised software application - Treatment 2, full intervention: all HEWs received a phone loaded with Frontline SMS application and two CHWs in village received mobile phones without the customised software application  Control group: No mobile phones were distributed Data analysis: Descriptive analyses | Critical appraisal tool used: CASP tool for randomised controlled trial.  Randomisation rigorous.  Blinding not done.  Participants managed rigorously.  Attrition low.  Data analysed according to groups. Groups and interventions comparable.  Context appropriate. Scores on appraisal tool: 9/11  Included  Level of evidence: II | Primary outcomes: In Treatment 1, ANC attendance increased from 15.8% to 31.5% and in Treatment 2, from 45.32% to 59.84%.  In control area there was a decrease, from 24.48% to 23.27%.  Home delivery in Treatments 1 and 2 reduced from 61.57% and 50.70%, to 33.73% and 35.82% respectively after the intervention.  Deliveries conducted in the presence of an HEW in Treatment 1 increased significantly, with a p value less than 0.050, whereas, in the control area there was a statistically significant reduction in deliveries attended by an HEW, at a p value less than 0.001.  Health institution delivery increased, from 23.44% to 55.07% in Treatment 1 compared to the control. This is a statistically significant difference, with p value <0.001.  Health-professional-assisted deliveries in the three groups increased, from 26.79%, 41.96% and 21.79% to 55.23%, 63.54% and 52.05% respectively.  No percentage change in stock-out of contraceptives at the health post level.  Full vaccination coverage decreased in all three groups, from 88.63%, 76.19% and 81.82%, to 58.31%, 58.72% and 62.98% respectively.  Timing of mother’s first PNC visit by CHW increased in the two intervention groups, from 80% and 68.30%, to 95% and 88.46% respectively. Secondary outcomes: Not stated or reported Limitations: Major change in the vaccination and contraceptive delivery processes across all health centers in the middle of the intervention.  Problems with the mobile phones and mobile network.  Logistical problems in availing timely project phones to the newly assigned health center heads and HEWs. Recommendations: Initiate systematic awareness programme on the potential of mHealth on health for the concerned stakeholders.  Scaling up mHealth use to other geographical areas and different health care applications. |
|  | Odeny TA, Bukusi EA, Cohen CR, Yuhas K, Camlin CS, McClelland, RS: **Texting improves testing: A randomized trial of two-way SMS to increase postpartum prevention of mother-to-child transmission retention and infant HIV testing**. *AIDS* 2014, **28**(15): 2307–2312. doi: 10.1097/QAD.0000000000000409 | Aim: To determine whether interactive text messages improved rates of clinic attendance and early infant HIV testing. Design: Randomised controlled trial Setting: Mix of rural and urban settings in Nyanza region, Kenya. Sample: Intervention group (n=195) and control group (n=93), total 388 Inclusion criteria: 18 years old.  Between 28 weeks gestation and delivery.  Enrolled in PMTCT.  Planning to remain in the study area.  Had access to a mobile phone.  Reported ability to read or had someone who could read SMS on their behalf and to whom they had disclosed their status. Exclusion criteria: Not stated | Primary outcomes: To improve maternal postpartum clinic attendance.  To improve virological infant HIV testing by 8 weeks postpartum. Secondary outcomes: Not stated Data collection: Biodata was collected during recruitment.  Women's return visits and infant HIV testing data were extracted from clinic records after the intervention. Intervention group: Up to 8 SMS were sent to women during pregnancy, from 28 weeks to delivery, and 6 SMS after delivery up to 6 weeks, by study staff who also collected data from records. Control group: No SMS, standard of care Data analysis: Unadjusted relative risk regression to compare the proportions of women retained and infants tested.  Kaplan-Meier methods and log-rank tests to compare time to clinic visit and time to infant HIV testing.  Unadjusted Cox  regression to estimate a hazard ratio comparing intervention to control arms outcomes. | Critical appraisal tool used: CASP tool for randomised controlled trial.  Randomisation rigorous.  Participants managed rigorously.  Attrition low.  Data analysed according to groups.  Groups and interventions comparable.  Context appropriate. Scores on appraisal tool: 10/11  Included  Level of evidence: II | Primary outcomes:Postpartum clinic visit: In SMS group, 38 of 194 (19.6%) women attended PNC, compared to 22 of 187 (11.8%) of the control group [relative risk (RR) 1.66, 95% confidence interval (CI) 1.02–2.70, p=0.04].  Women in the SMS arm had a significantly higher probability of attending clinics within 8 weeks, compared to those in the control arm [RR 1.83, 95% CI 1.11–3.01]. Infants DBS testing: In the SMS group, 172 of 187 (92.0%) were tested, compared to 154 of 181 (85.1%) in the control group [RR 1.08, 95% CI 1.00–1.16, p=0.04].  The probability of infant HIV testing within 8 weeks was significantly higher in the SMS than in the control group [RR 1.09, 95% CI 1.01–1.17]. Secondary outcomes: Not stated or reported Limitations: Exclusion of women who lacked access to phones, or who shared phones but had not disclosed their HIV status. Recommendations: Future qualitative “exit” interviews to provide insight into perceptions of the messages, potentially illuminating their mechanism of action.  Expansion of such affordable and easily accessible mHealth intervention. |
|  | Bigna JJR, Noubiap JJN, Kouanfack C, Plottel CS, Koulla-shiro S: **Effect of mobile phone reminders on follow-up medical care of children exposed to or infected with HIV in Cameroon (MORE CARE): A multicentre , single-blind , factorial , randomised controlled trial.** *Lancet Infect Dis* 2014, **14**(7):600–6008. doi: 10.1016/S1473-3099(14)70741-8. | Aim: To assess whether reminders sent to carers by text message, mobile phone call, or concomitant text message and mobile phone call, increased attendance at medical appointments for HIV care.  To ascertain the most efficient (i.e., cost-effective) method of mobile-phone-based reminder. Design: Randomised controlled trial Setting: Three regions in Cameroon representing urban (Essos), semi-urban (Kousséri), and rural (Goulfey) settings. Sample: 224 participants per group Inclusion criteria: Aged 18 years or older.  Accompanying a child younger than 15 years who was infected with or had been exposed to HIV for HIV care.  Owned a mobile phone.  Able to communicate verbally, read or write in English or French. Exclusion criteria: Did not have a mobile phone.  Had an appointment scheduled in less than 3 days.  Could not communicate verbally.  Could not use written communication.  Did not want to receive a text message.  Did not want to receive a phone call. | Primary outcomes: To improve attendance to the previously scheduled appointment for HIV care.  To improve primary efficacy outcome relative to the staff working time used and the direct financial costs of the intervention. Secondary outcomes: To report success or failure of each reminder. Data collection: At recruitment, study nurse collected:   - Participant identification code; - The date of the next scheduled follow-up appointment; - The treating physician’s name; - The participant’s phone number; and - The preferred language of communication (French or English).   During intervention the contents of the text messages, phone calls and procedures were documented. Intervention group: Study medical administrative assistant implemented three interventions to mothers to provide an appointment reminder by   - Text message; - mobile phone call; or - Text messages and phone calls.   The content of the text messages, phone calls and procedures were documented for analysis after intervention. Control group: No reminder (usual practice) Data analysis: Descriptive statistics.  Odds ratios (ORs) with 95% CIs to compare the efficacy of the different interventions with respect to the primary efficacy outcome.  t test to compare continuous variables.  χ² test to compare binary variables.  Multivariate regression analyses.  Hosmer and Lemeshow test of goodness of fit. | Critical appraisal tool used: CASP tool for randomised controlled trial.  Randomisation rigorous.  Group not similar at start of trial.  Participants managed rigorously.  Attrition low.  Data analysed according to groups.  Groups and interventions comparable.  Context appropriate. Scores on appraisal tool: 9/11  Included  Level of evidence: II | Primary outcomes: All interventions improved attendance compared to control:  For text message plus call the OR was 8.5 (2.8–25.2; p<0.0001).  For call 5.7 (2.2–14.5; p<0.001).  For text message 3.0 (1.3–6.8; p=0·01).  The Hosmer and Lemeshow goodness-of fit test generated a χ² of 8.7  (df=8; p=0.37).  Text messaging was the most efficient intervention when both the direct costs of the intervention and staff working time were taken into account.  When only the direct costs of the intervention were taken into account, the phone call was the most efficient intervention.  When all text messages and calls (i.e., across all relevant groups) were taken together, the staff working time did not differ significantly between the text message and call interventions. Secondary outcomes: Not reported. Limitations: No guarantee that the message was actually read by the participant.  Sample size had a low statistical power for detecting differences between the intervention groups.  Measure of staff working time did not take into consideration factors that could indirectly affect the time taken to implement each intervention, such as documentation. Recommendations: Assess the acceptability of these reminder methods for carers and patients before widespread implementation. |
| Quasi-experimental studies (n=4) | | | | | |
|  | Prinja S, Nimesh R, Gupta A, Bahuguna P, Gupta M, Thakur JS: **Impact of m-health application used by community health volunteers on improving utilisation of maternal, new-born and child health care services in a rural area of Uttar Pradesh, India.** *Trop Med Int Heal* 2017, **22** (7):895-907. doi: 10.1111/tmi.12895 | Aim: To assess the impact of an mHealth intervention used by community health volunteers on uptake of maternal, neonatal and child health (MNCH) services. Design: Quasi-experimental study Setting: Community development blocks of Kaushambi district of rural area of Uttar Pradesh, India. Sample:Pre-intervention: 450 mothers with children aged 29 days to 6 months, and 310 women with children aged 12–23 months. Post-intervention: 534 women with children aged 29 days to 6 months.  1 019 women with children between 12 and 23 months of age. Inclusion criteria: Women with children aged 29 days to 6 months.  Women with children aged 12–23 months. Exclusion criteria Not indicated | Primary outcome: To improve coverage of key MNCH services such as   - ANC visits; - Consumption of iron-folic acid (IFA) supplementation; - Tetanus toxoid vaccine; and - Full antenatal care.  Secondary outcomes: To improve quality of antenatal care using the following indicators:   - Measurement of height; - Measurement of weight; - Measurement of blood pressure; and - A blood and urine test.   To improve identification and reporting of complications during pregnancy and after childbirth.  To improve coverage of   - Institutional deliveries - Ambulance usage for delivery; and - Full immunisation.  Data collection: Annual Health Survey individual-level data was accessed from Ministry of Health and Family Welfare in 2011 before the intervention.  Household survey was done in 2015 by a team of 35 graduate-level field investigators who collected data from mothers on:   - Utilisation of antenatal care services; - Institutional delivery; - Postnatal care; and - Immunisation status of infants.  Intervention group: Community health volunteers (ASHAs) used mHealth application as job aids to support,   - Client assessment; - Counselling; - Early identification; and - Treatment and/or rapid referral of pregnancy, postpartum and newborn complications.  Control group: No mHealth intervention Data analysis: Matched analysis using Nearest neighbour method with 1:1 algorithm and calliper width of 0.025 to detect differences in the two groups.  Difference-in-difference analysis (DID) to compare changes in coverage of services in intervention area relative to the control area was used. | Critical appraisal tool used: Joanna Briggs Institute (JBI) Critical Appraisal Checklist for quasi experimental studies.  Clear cause and effect described.  Participants, groups and intervention comparable.  Follow-up complete and data analysed according to the groups.  Multiple measurements of outcome present.  Measurement of outcome reliable with appropriate statistics. Score on appraisal: 9/9  Included  Level of evidence: I | Primary outcomes: Coverage of ≥3 ANC visits increased by 10.3%.  IFA supplementation coverage increased by 12.58%.  Coverage of ≥2 tetanus toxoid increased by 4.28%  Full ANC increased by 1.1%. Secondary outcomes: There was decrease in weight taking (10.7%) and blood testing (5.7%).  There was increase in blood pressure by 0.7 %, and urine testing by 2.4%.  Self-reporting of illnesses/complication during pregnancy increased by 13.11%, and by 19.6 after delivery.  Institutional delivery increased by 4.02%.  Ambulance usage increased by 2.06%.  Full immunisation decreased by 6.4%. Limitations: Allocation of interventions was not randomised.  Limited number of variables used for matching.  Sample of women dropped after matching in pre-study.  Different reference period used for pre-and post-study. Recommendations: An assessment of the costs to understand whether the improvements in service utilisation justifies the increase in investments on account of mHealth.  Replication of such an intervention in the entire state or country. |
|  | Prieto JT, Zuleta C, Rodríguez JT: **Modeling and testing maternal and newborn care mHealth interventions: A pilot impact evaluation and follow-up qualitative study in Guatemala.** *J Am Med Informatics Assoc* 2017, **24**:352-60. doi: 10.1093/jamia/ocw102 | Aim: To develop a descriptive model of structural characteristics of mHealth in the context of newborn nutrition, and to assess the effects of illustrative interventions. Design: Experimental design Setting: Rural clinics in Guatemala Sample: 100 women Inclusion criteria: Mothers more than 8 months pregnant or who had babies not older than 4 months.  Understand written Spanish.  Willing to participate in a mobile technology programme. Exclusion criteria: Not stated | Primary outcomes: To compare the effects of simultaneous interventions on knowledge and self-reported health behaviour regarding newborn nutrition among new mothers. Secondary outcomes: Not stated Data collection: Face-to-face interviews were conducted  Women were interviewed and asked about their knowledge and practices of breastfeeding at recruitment and at the end of the study, by two investigators. Intervention group: There were three groups with different interventions:   - Group 1 received text messages twice a week. - Group 2 were assigned to 1 of 3 peer-to-peer groups of 10 individuals each. - Group 3 were assigned to 1 of 3 peer-to-peer groups (as for Group 2), received information regarding breastfeeding practices (as for Group 1) and could communicate with a health professional.   Mothers received text messages twice a week for 23 weeks, related to newborn nutrition, on mobile phones they had been given when they enrolled in the project**.** Control group: Mothers simply given a mobile phone and were instructed to use it for matters related to their babies. Data analysis: Descriptive statistics | Critical appraisal tool used: JBI Critical Appraisal Checklist for quasi-experimental studies.  Clear cause and effect described.  Groups and intervention comparable.  Data analysed according to the groups.  Multiple measurements of outcome present.  Appropriate statistics. Scores on appraisal tool: 7/9  Included  Level of evidence: II | Primary outcomes: There was increase in knowledge, from 58% before intervention, to 93% after intervention. This was a significant change (p<.001)  The most effective intervention in terms of increase in knowledge was Group 1 that received text messages only.  Most participants in Group 1 (60%, n=12/20) experienced a knowledge increase.  All individuals in Group 1 (100%, n=20/20) were, at the end of intervention, aware of the exclusive breastfeeding message, compared to 67% (n=8/12) in the control group.  No significant difference in self-reported behaviour, but a significant relation (p=0.010) between changes in knowledge and changes in self-reported behaviour was found.  89% (n=54/61) of those participants who were aware of the exclusive breastfeeding message at the end of the experiment reported to have exclusively breastfed their babies. Secondary outcomes: Not indicated Limitations: Not stated Recommendations: New mHealth studies should explore the potential of mixed methodologies for the analysis of low-resource health environments.  They should also exploit the possibilities of innovative data collection mechanisms.  Future projects could consider finding financial equilibrium by asking for a subscription fee to cover the cost of text message transmission. |
|  | Shiferaw S, Spigt M, Tekie M, Abdullah M, Fantahun M, Dinant GJ: **The effects of a locally developed mHealth intervention on delivery and postnatal care utilization; A prospective controlled evaluation among health centres in Ethiopia.** *PLoS One* 2016, **11**:1–15. doi: 10.1371/journal.pone.0158600 | Aim: To determine whether an mHealth intervention and training of health providers on client-centred care can improve maternity service utilisation, specifically repeat ANC attendance, institutional delivery, and PNC service utilisation, compared to the conventional approach. Design: Quasi-experimental study Setting: Semen Shewa Zone, Amhara region, central Ethiopia. Sample: 10 health facilities (5 intervention, 5 control). A sample size of 500 in each group. Inclusion criteria: Not stated Exclusion criteria: Not stated | Primary outcomes: To improve percentage of women who had   - At least 4 ANC visits; - Institutional delivery; and - PNC visits at the health centre.  Secondary outcomes: Not stated Data collection: Exit interviews were conducted by health workers (nurses/health officers) with consecutive ANC attendants before and after intervention.  Data collected before was biodata.  After the intervention, data was collected through facility record review and exit interviews, on   - Attendance of at least 4 ANC visits; - Institutional delivery; and - PNC visit within 6 hours of delivery.  Intervention group: Health workers received an Android phone (3 phones per facility) loaded with an application that sends reminders for scheduled visits during ANC,  delivery and PNC, and educational messages on danger signs and common complaints during pregnancy. Control group: No mobile phone and no reminders (usual care). Data analysis: Chi-square tests to compare the baseline and follow-up characteristics of clients.  Logistic regression models.  95% CI for the difference of proportions.  Significance set at α=0.05. | Critical appraisal tool used: JBI Critical Appraisal Checklist for quasi-experimental studies.  Clear cause and effect described.  Participants, groups and intervention comparable.  Data analysed according to the groups.  Multiple measurements of outcome present.  Measurement of outcome reliable with appropriate statistics. Scores on appraisal tool: 8/9  Included  Level of evidence: II | Primary outcomes: At least 4 antenatal visits (27.0% in intervention versus 23.4% in control); AOR: 1.31(95% CI  1.00–1.72) but not statistically significant.  Institutional delivery (43.1% in intervention versus 28.4% in control); AOR: 1.98 (95% CI 1.53–2.55).  PNC in the health centres (41.2% in the intervention versus 21.1% in control); AOR: 2.77 (95% CI 2.12–3.61). Secondary outcomes: Not stated Limitations: Limited background information about women in the medical records. Recommendations: Explore the possible mechanisms of action of various components of existing mobile health solutions and evaluate their effectiveness in other priority health programmes, such as malnutrition, family planning and immunisation of children on a bigger scale.  Provide more applications that offer practical solutions to health challenges that are likely to benefit from technology-based innovations. |
|  | Uddin MJ, Shamsuzzaman, M, Horng, L, Labrique, A, Vasudevan L, Zeller K, Chowdhury M, Larson CP, Bishai D, Alam N: **Use of mobile phones for improving vaccination coverage among children living in rural hard-to-reach areas and urban streets of Bangladesh**. *Vaccine* 2016, **34**, 276-283. doi: 10.1016/j.vaccine.2015.11.024 | Aim: To develop and test a mechanism to use mobile phones to improve child vaccination coverage.  To assess feasibility and effectiveness of mobile phone system of vaccination registry, newborn tracking, and parental reminders. Design: Quasi experiment Setting: Rural hard-to-reach and urban street-dweller populations of Bangladesh. Sample: 520 per group Inclusion criteria: Over 18 years old.  Had given birth within one year prior to data collection.  Able to give written informed consent in Bengali.  Children age 0–11 months. Exclusion criteria: Not stated | Primary outcomes: To compare vaccination coverage among 0–11-month-old children in rural hard-to-reach and urban street-dweller areas before and after mobile intervention. Secondary outcomes: Not indicated Data collection: EPI cards, when available, maternal recall with structured questionnaires where cards were not available Intervention group: Mothers were sent automatic SMS reminders about upcoming EPI sessions. Control group: No intervention, usual care Data analysis: Chi-square tests to compare sociodemographic characteristics.  Z-tests to compare vaccination coverage between baseline and endline surveys.  Difference-in-difference (DID) estimation was used to track longitudinal differences in coverage from baseline to end line between control versus intervention areas.  Logistic regression model to test DID for statistical significance and a 95% confidence interval around the odds ratio (OR). | Critical appraisal tool used: JBI Critical Appraisal Checklist for quasi-experimental studies.  Clear cause and effect described.  Participants, groups and intervention comparable.  Follow-up complete and data analysed according to the groups.  Multiple measurements of outcome present.  Measurement of outcome not similar.  Appropriate statistics used. Score on appraisal: 8/9  Included  Level of evidence: II | Primary outcomes: Full vaccination in the rural intervention area increased, from 58.9% to 76.8%, while decreasing in the rural control area, from 65.9% to 55.2%, resulting in a DID of +29.5% (p<0.001).  Full vaccination rates in the urban intervention area increased from 40.7% to 57.1%, while decreasing in the urban control area from 44.5% to 33.9%,  resulting in a DID of +27.1% (p<0·05).  Intervention effect on age-appropriate vaccination was positive for all age groups, with DIDs ranging from +13.1% to +30.5% and ORs ranging from 2.5 to 4.6 (p<0.001 across all rural versus urban comparisons per age group).  The largest intervention effect was on age-appropriate vaccination for children over 70 days, OR 4.6 in the urban intervention area (p<0.001, 95% CI 2.1–7.8). Limitations: Time and funding constraints.  Using maternal recall for vaccination history was not as accurate as EPI cards, but mothers could not produce the cards. Recommendations: Research in mechanisms to increase the mHealth intervention’s health impacts, scalability, sustainability, and cost-effectiveness**.**  Use of the mHealth in low-income countries with high mobile phone use and robust EPI programmes. |
| Cross-sectional surveys(n=2) | | | | | |
|  | Alam M, D’Este C, Banwell C, Lokuge K: **The impact of mobile phone based messages on maternal and child healthcare behaviour: A retrospective cross-sectional survey in Bangladesh**. *BMC Health Serv Res* 2017, **17**:1-13. doi: 10.1186/s12913-017-2361-6. | Aim: To evaluate the effectiveness of a mobile-phone-based intervention in Bangladesh, designed to improve compliance with WHO guidelines related to delivery and neonatal care. Design: Cross-sectional survey Setting: Five districts in Bangladesh. Sample: 476 mothers Inclusion criteria: Exposed to the service for at least 3 months during pregnancy or after childbirth.  Live birth in the last delivery.  Last born child’s age between 3 and 18 months. Exclusion criteria: Could not be contacted by phone for an appointment.  Not at home during the interview.  Unwillingness to participate.  Outside the survey areas for an indefinite time. | Primary outcomes: To improve assistance during delivery. Secondary outcomes: To improve breastfeeding immediately post birth.  To improve delayed bath of newborn up to 3 days.  To improve attendance of four PNC visits. Data collection: Involved face-to-face interviews in participants’ homes by two researchers.  Data was collected on:   - Where the baby was born; - Who assisted at delivery; - What was baby fed right after birth; - When was baby given first bath after birth; and - Frequency of PNC visits.  Data analysis: Bivariate analysis to compare characteristics between exposure groups.  Chi-square tests to examine associations between explanatory and outcome variables.  Multiple regression analyses to investigate the relationship between exposure to early warning messages and the outcomes of interest.  Multinomial regression analysis for assistance during delivery and timing of first bath.  Logistic regression analysis for breastfeeding immediately post birth.  Negative binomial regression for frequency of PNC visits. | Critical appraisal tool used: CASP tool for cross-sectional studies.  Focus clear.  Methods appropriate.  Recruitment not described.  Accurate measures used.  Data collection, presentation and analysis acceptable.  Clear statement of the findings.  Context appropriate. Scores on appraisal tool: 9/11  Included  Level of evidence: II | Primary outcomes: 307 (65%) women assisted by trained personnel during delivery at health facility.  111 (23%) women had a skilled birth attendant present at birth.  58 (12%) were assisted by untrained relatives or local traditional birth attendants at home deliveries.  These findings were not statistically significant. Secondary outcomes: 443 (93%) fed their babies colostrum immediately after birth.  33 (7%) fed sweetened water, honey or mustard oil. This had no significant association.  294 (62%) women bathed their babies 72 h or more after delivery,  80 (17%) bathed babies in the first 48 h.  100 (21%) bathed babies between 48 and 72 h.  Findings about first bath were significant (RRR 1.7; 95% CI 0.93–3.0;  p=0.083)  273 (57%) did not have PNC visits within 42 days after childbirth.  The rest reported having 1 (n=79, 17%), 2 (n=54, 11%), 3 (n=34, 7%) or 4 visits (n=36, 8%). This is not statistically significant (IRR: 1.2; 95%CI 0.94–1.6; p=0.117). Limitations: Limited generalisability.  Association between early messages and outcomes limited.  Power of study is based on a moderate to large association.  Reliance on self-report of practices, which can introduce bias and problems of recall.  Many contextual factors, which contribute to delivery practices, not captured. Recommendations: Need for larger studies and randomised designs to overcome the limitations encountered in this study. |
|  | Sutcliffe CG, Thuma PE, van Dijk JH, Sinywimaanzi K, Mweetwa S, Hamahuwa M, et al.: **Use of mobile phones and text messaging to decrease the turnaround time for early infant HIV diagnosis and notification in rural Zambia: An observational study.** *BMC Pediatr* 2017, **17**(66):1–10. doi: 10.1186/s12887-017-0822-z | Aim: To evaluate the feasibility of using:   - Mobile phones to contact mothers in rural Zambia; and - SMS reporting system to deliver test results.  Design: Cross-sectional survey Setting: Macha Hospital in a rural area of Choma District, Southern Province, Zambia. Sample: 419 mother-infant pairs Inclusion criteria: Bringing infants to either the HIV clinic or a primary health centre associated with the hospital.  Access to a mobile phone.  Willingness to participate Exclusion criteria: Not stated | Primary outcome: To improve the process of early infant diagnosis using text or phone calls. Secondary outcome: To evaluate the performance of a national SMS reporting system to deliver test results from the central laboratory to the clinic. Data collection: Clinic and medical records were retrieved by study nurse.  Data was collected on time when the mother returned to receive her child’s test results. Data analysis: Descriptive statistics.  Chi-square tests to compare categorical variables.  Wilcoxon rank sum tests to compare continuous variables. | Critical appraisal tool used: CASP tool for cross-sectional studies.  Focus clear.  Methods appropriate.  Recruitment not clear.  Accurate measures used.  Data collection not clear.  Data presentation and analysis acceptable.  Clear statement of the findings.  Context appropriate. Scores on appraisal tool: 7/11  Included  Level of evidence: IV | Primary outcomes: 87% mothers preferred a phone call above text messages.  Contact made with mother in 96% of tests.  Median of 6 days after receiving test results before mother was called.  Mother visited clinic after having been asked to attend clinic: median 18 days, IQR 5,40.  Shorter duration of returning to clinic lead to early diagnosis into care. Secondary outcomes: Rural health centres were first called median 7 days (IQR 3, 20).  Median time from sample collection to ART initiation 103 days (IQR 71,144).  Median time from delivery of test results to clinic to disclosure to mother 37 days (IQR 17,57).  Mothers with infants with positive results returned to the clinic sooner (median 15 days; IQR 7,27) than those with negative results (median 39 days IQR 18,61; p<0.0001).  SMS reporting improved from 38% (2013) to 91% (2014). Limitations: Not possible to determine the impact of the intervention on retention.  Evaluation of the national SMS system did not involve the central laboratory or the individuals involved in sending the text messages.  Not able to determine the full benefit of mobile technology in reducing the turnaround time from the lab to the clinic. Recommendations: Additional resources necessary for ongoing monitoring to ensure quality.  Programmes and clinics need to weigh the costs of text message systems against the benefits of earlier diagnosis and treatment for HIV-infected infants. |
| Qualitative studies (n=1) | | | | | |
|  | Ayiasi RM, Atuyambe LM, Kiguli J, Orach CG, Kolsteren P, Criel B: **Use of mobile phone consultations during home visits by community health workers for maternal and newborn care: Community experiences from Masindi and Kiryandongo districts, Uganda.** *BMC Public Health* 2015, **15**:560. | Aim: To explore perceived maternal and newborn benefits of VHTs making home visits to prenatal and postnatal women and using mobile phone consultations to link VHTs to professional health care workers for further advice. Design: Qualitative design Setting: Two districts in Uganda Sample: 67 interviews (with women, VHTS, professional health workers)  In total 87 participants | Outcomes: To determine:   - The perceived benefits of home visits and phone consultations; - Perceptions regarding recommended newborn care practices; - Perceptions regarding delegation of promotional maternal and newborn messages to VHTs.  In depth interview: Conducted with prenatal, postnatal women, VHT members and professional health workers. Data collection: Through in-depth interviews, key informant interviews and group discussions. Data analysis: Interviews were audiotaped and transcribed directly into English.  Typed texts were read several times and exported to NVivo Version 10.  Latent content analysis was done (subjective interpretation of text  data through systematic classification process of coding and identifying themes or patterns). | Critical appraisal tool used: CASP tool for qualitative research.  Clear statement of aims.  Methodology appropriate.  Design, recruitment and data collection appropriate to aims.  Researcher-participant relationship adequately addressed.  Ethical issues not adequately addressed.  Data analysis rigorous.  Clear statements of findings.  Context appropriate. Scores on appraisal tool: 9/10  Included  Level of evidence: II | Outcomes: Perceived benefits of home visits and phone consultations,   - Improved access to maternal and newborn care; - Provided additional information leading to attitudinal change; - Contributed to emotional satisfaction among VHTs, prenatal and postnatal women; and - Elevated social status for VHTs, women felt well cared for and enlisted male partner support.   Perception regarding recommended newborn care practices was as follows:   - Cord care: Nearly all women accepted and practised non-application of substances on the umbilical stump. Women reported they relied on advice offered by their VHTs; - Thermal care: About 3 in 4 women admitted to practising delayed bathing; following the VHT’s teachings; and - Breastfeeding and pre-lacteal feeds: Nearly all women agreed to initiate breastfeeding within one hour after delivery. They learnt from VHTs that newborns were too young to take in anything other than breastmilk.   Perceptions regarding delegation of promotional maternal and newborn messages to VHTs were that promotional maternal and newborn interventions to VHTs were mostly accepted. Working with VHTs was found acceptable. Limitations: Related to qualitative technique. Limited number of women. Recommendations: Not stated |
