# Supplementary material for: mHealth communication to strengthen postnatal care in rural areas: a systematic review
Source: BMC Pregnancy Childbirth. 2019 Nov 6;19:406. doi: 10.1186/s12884-019-2531-0 (PMC6836428; doi:10.1186/s12884-019-2531-0)
Supplement: Supplementary file 2 — Additional file 2. Data synthesis on how mHealth strengthens PNC in a rural areas. This file highlights the types of mHealth communication and the determinants of PNC uptake. [file 12884_2019_2531_MOESM2_ESM.docx]

**Additional file 2: Data synthesis** **on how mHealth strengthen PNC in a rural area.**

| **Article** | **Type of mHealth** | **Strengthening of PNC** | | | | |
| --- | --- | --- | --- | --- | --- | --- |
|  |  | **Intention** | **Skills** | | **Environment constraints/facilitators** | |
|  |  | **Attitudes** | **Norms** | **Self-efficacy** |  |  |
| Ayiasi RM, Kolsteren P, Batwala V, Criel B: **Effect of village health team home visits and mobile phone consultations on maternal and newborn care practices in Masindi and Kiryandongo, Uganda : A community-intervention trial**. *PLoS Med* 2016 **11**(4):e0153051. doi: 10.1371/journal.pone.0153051  **LEVEL OF EVIDENCE: II** | Mobile phone call consultations by village health teams with healthcare professionals. | Not addressed | Village health team members used mobile phone calls to positively influence mothers to deliver in hospitals and seek care for the newborns’ illnesses. | Not addressed | Use of mobile phone consultation with healthcare professionals positively influenced mothers to improve cord and thermal care of their newborns. | Healthcare professionals in consultation with village health team members facilitated timely and prompt seeking of maternal and newborn care. |
| Atnafu A, Otto K, Herbst CH. **The role of mHealth intervention on maternal and child health service delivery: findings from a randomized controlled field trial in rural Ethiopia**. *mHealth* 2017, 3:39-39. doi: 10.21037/mhealth.2017.08.04  **LEVEL OF EVIDENCE: II** | One-way mobile phone messaging to health extension workers and voluntary community health workers. | Not addressed | Health extension workers and voluntary community health workers used one way mobile phone messaging to positively influence mothers to deliver in hospitals.  Health extension workers used one-way mobile phone messaging to positively influence mothers to deliver in their presence. | Not addressed | Not addressed | Use of mobile phone messaging by community health workers facilitated mothers to receive PNC services soon after birth. |
| Odeny TA, Bukusi EA, Cohen CR, Yuhas K, Camlin CS, McClelland RS: **Texting improves testing: A randomized trial of two-way SMS to increase postpartum prevention of mother-to-child transmission retention and infant HIV testing.** *AIDS* 2014, **28**(15):2307–2312. doi: 10.1097/QAD.0000000000000409  **LEVEL OF EVIDENCE: I** | Interactive mobile phone messaging between postnatal mothers and study nurse. | Use of text messages positively influenced mothers to attend postnatal clinics.  Use of text messages positively influenced mothers to take their infants to the clinic for HIV testing. | Not addressed | Not addressed | Not addressed | Use of interactive mobile phone messages facilitated postnatal mothers to receive postnatal care services that were personal. |
| Bigna JJR, Noubiap JJN, Kouanfack C, Plottel CS, Koulla-shiro S: **Effect of mobile phone reminders on follow-up medical care of children exposed to or infected with HIV in Cameroon (MORE CARE ): A multicentre , single-blind , factorial , randomised controlled trial.** *Lancet Infect Dis* 2014, **14**(7):600–6008. doi: 10.1016/S1473-3099(14)70741-8.  **LEVEL OF EVIDENCE:II** | One-way mobile phone messaging appointment reminders by study nurse to mothers.  Mobile phone call appointment reminders by study nurse to mothers.  Combined mobile phone messaging and mobile phone calls for appointment reminders by study nurse to mothers. | Not addressed | Not addressed | One-way mobile phone messaging and mobile phone calls positively influenced attendance of scheduled appointments for HIV care by mothers. | Not addressed | Not addressed |
| Prinja S, Nimesh R, Gupta A, Bahuguna P, Gupta M, Thakur JS. **Impact of m-health application used by community health volunteers on improving utilisation of maternal, new-born and child health care services in a rural area of Uttar Pradesh, India.** *Trop Med Int Heal* 2017, **22** (7):895-907. doi: 10.1111/tmi.12895  **LEVEL OF EVIDENCE: I** | One-way mobile phone messaging to community health volunteers.  Mobile phone audio-visual application used by community health volunteers as job aids. | Not addressed | Community health volunteers used mobile phone audio-visual to positively influence mothers to deliver in the hospital. | Mobile phone audio-visual application by community health volunteers positively influenced mothers to recognise and report compli­cations after pregnancy. | Not addressed | Timely one-way mobile phone messaging reminders to community health volunteers facilitated mothers to receive PNC services and facilitated greater contact with public sector. |
| Prieto JT, Zuleta C, Rodríguez JT: **Modeling and testing maternal and newborn care mHealth interventions: A pilot impact evaluation and follow-up qualitative study in Guatemala.** *J Am Med Informatics Assoc* 2017, **24**:352-360. doi: 10.1093/jamia/ocw102  **LEVEL OF EVIDENCE: II** | One-way mobile phone messaging to mothers on newborn nutrition by health professionals. | Use of mobile phone messaging on nutrition positively influenced mothers to believe in the value of exclusive breast_­_feeding. | Not addressed | Use of mobile phone messaging on nutrition positively influenced mothers to exclusively breastfeed their newborns. | Use of mobile phone messaging on nutrition positively influenced mothers to practice exclusive breastfeeding. | Not addressed |
| Shiferaw S, Spigt M, Tekie M, Abdullah M, Fantahun M, Dinant GJ: **The effects of a locally developed mHealth intervention on delivery and postnatal care utilization; A prospective controlled evaluation among health centres in Ethiopia.** *PLoS One* 2016, **11**:1–15. doi: 10.1371/journal.pone.0158600  **LEVEL OF EVIDENCE: II** | One-way mobile phone messaging reminder to the health worker and phone call reminder to the mother. | One-way mobile phone messaging reminder to the health worker and phone call reminder to the mother positively influenced mother to deliver in the hospital and seek PNC services in the health centres. | Not addressed | One-way mobile phone messaging reminder to the health worker and phone call reminder to the mother positively influenced mothers to feel valued and responsive to following advice. | Not addressed | One-way mobile phone messaging reminder to the health worker facilitated tracking of the mothers, reminding them to attend to PNC services. |
| Uddin MJ, Shamsuzzaman M, Horng L, Labrique A, Vasudevan L, Zeller K, et al.: **Use of mobile phones for improving vaccination coverage among children living in rural hard-to-reach areas and urban streets of Bangladesh.** *PMC* 2017, **34**:276-83. doi: 10.1016/j.vaccine.2015.11.024  **LEVEL OF EVIDENCE: II** | One-way mobile phone messaging to both the mothers and health workers. | One-way mobile phone messaging to both the mothers and health workers positively influenced mothers to vaccinate their children in a timely manner. | Not addressed | Not addressed | Not addressed | One-way mobile phone messaging to both the mothers and health workers facilitated timely vaccination of children. |
| Alam M, D’Este C, Banwell C, Lokuge K: **The impact of mobile phone based messages on maternal and child healthcare behaviour: A retrospective cross-sectional survey in Bangladesh**. *BMC Health Serv Res* 2017, **17**:1-13. doi: 10.1186/s12913-017-2361-6.  **LEVEL OF EVIDENCE: II** | One-way mobile phone text or voice messages to mothers on delivery and neonatal care. | One-way mobile phone text or voice messages positively influenced mothers to change their beliefs on delaying first bath, and breastfeeding their babies with colostrum immediately after birth. | Not addressed | Not addressed | One-way mobile phone text or voice messages positively influenced mothers to breastfeed their babies immediately after birth and bath their newborn only after three days. | Not addressed |
| Sutcliffe CG, Thuma PE, van Dijk JH, Sinywimaanzi K, Mweetwa S, Hamahuwa M, et al.: **Use of mobile phones and text messaging to decrease the turnaround time for early infant HIV diagnosis and notification in rural Zambia: An observational study.** *BMC Pediatr* 2017, **17**(66):1-10. doi: 10.1186/s12887-017-0822-z  **LEVEL OF EVIDENCE: IV** | One-way mobile phone messaging or phone calls to mothers to inform of them of test results. | Not addressed | Not addressed | Not addressed | Not addressed | One-way mobile phone messaging or phone calls facilitated mothers to return to the clinic, leading to early diagnosis and enrolment for care. |
| Ayiasi RM, Atuyambe LM, Kiguli J, Orach CG, Kolsteren P, Criel B: **Use of mobile phone consultations during home visits by community health workers for maternal and newborn care: Community experiences from Masindi and Kiryandongo districts, Uganda.** *BMC Public Health* 2015, **15**:560.  **LEVEL OF EVIDENCE: II** | Mobile phone call consultations by village health teams with health care professionals. | Mobile phone call consultation by village health teams with health care professionals positively influenced mothers to change beliefs in newborn care practices, i.e., breastfeeding immediately after birth, cord care and thermal care.  The intervention also lead to attitudinal change by providing additional information. | Mobile phone call consultation by village health teams with health care professionals positively influenced male partner participation in maternal and newborn care issues. | Mobile phone call consultation by village health teams with health care professionals positively influenced mothers’ confidence to arrive at more favourable outcomes for their babies. | Mobile phone call consultation by village health teams with health care professionals positively influenced mothers to achieve desired newborn care practices, i.e., breastfeeding immediately after birth, cord care and thermal care. | Mobile phone call consultation by village health teams with health care professionals facilitated mothers’ access to maternal and newborn care services. |

**Notes:**

- **Type of mHealth**: Device + manner of message conveyed.
- **Norms**: Who is “social network’? + What is outcome of their influence?
- **Attitude**: Change in behaviour. (Behavioural attitude. The synonyms are beliefs, views, act, approach, perspective, position, stand, expression, mindset, inclination.)
- **Efficacy**: The ability to produce a desired or intended result. (Synonyms are effectiveness, effectualness, efficaciousness, efficacity, efficiency, productiveness, ability, capability, capacity. potency, puissance, strength.)
- **Skill**: A particular ability. (Synonyms are expertise, skilfulness, expertness, adeptness, adroitness, deftness, dexterity, ability, prowess, mastery, competence, competency, capability, efficiency, aptitude, artistry, art, finesse, flair, virtuosity, experience, professionalism, talent, cleverness, smartness, ingenuity, versatility, knack, readiness, handiness.)
- **Environmental constraints/facilitators**: Physical hindrances/enablers.
